# *Sporidesmiellalignicola* sp. nov., a new hyphomycetous fungus from freshwater habitats in China

**DOI:** 10.3897/BDJ.9.e77414

**Published:** 2021-12-15

**Authors:** Xiao-Hong Li, Yu-Lin Liu, Hai-Yan Song, Dian-Ming Hu, Yang Gao, Hai-Jing Hu, Jian-Ping Zhou

**Affiliations:** 1 Bioengineering and Technological Research Centre for Edible and Medicinal Fungi, Jiangxi Agricultural University, Nanchang, China Bioengineering and Technological Research Centre for Edible and Medicinal Fungi, Jiangxi Agricultural University Nanchang China; 2 Jiangxi Environmental Engineering Vocational College, Ganzhou, China Jiangxi Environmental Engineering Vocational College Ganzhou China; 3 Key Laboratory of Crop Physiology, Ecology and Genetic Breeding (Jiangxi Agricultural University), Ministry of Education of the P.R. China, Nanchang, China Key Laboratory of Crop Physiology, Ecology and Genetic Breeding (Jiangxi Agricultural University), Ministry of Education of the P.R. China Nanchang China; 4 College of Bioscience and Bioengineering, Jiangxi Agricultural University, Nanchang, China College of Bioscience and Bioengineering, Jiangxi Agricultural University Nanchang China

**Keywords:** freshwater fungi, lignicolous fungi, hyphomycetes, taxonomy

## Abstract

**Background:**

Freshwater fungi, growing on submerged wood, can promote the degradation of organisms and the reuse of rotten wood energy and play key roles in freshwater ecosystems. Here, a new hyphomycetous fungus, *Sporidesmiellalignicola*, was isolated and identified from submerged wood samples collected in a small stream in Jiangxi Province, south-eastern China.

**New information:**

The new taxon was studied, based on morphological characters and phylogenetic analyses combined with LSU, ITS, *TEF1α* and *RPB2* sequences data. *Sporidesmiellalignicola* was morphologically characterised by its pink colonies on PDA medium in prophase, macronematous, mononematous, solitary, brown, septate, unbranched, straight or slightly flexuous conidiophores with holoblastic, polyblastic, integrated, terminal, pale brown conidiogenous cells and cylindrical, narrowly clavate, broadly obovoid to cuneiform, 3–4-distoseptate, olivaceous brown or brown conidia with rounded apex. A phylogenetic tree was constructed, based on the combination of LSU, ITS, *TEF1α* and *RPB2* sequences data.

## Introduction

The genus *Sporidesmiella* was introduced by Kirk to accommodate two newly-described species and four new combinations from *Sporidesmium*, with *Sporidesmiellaclaviformis* as the type species (Kirk 1982). *Sporidesmiella* was commonly characterised by having clavate or obovoid to cuneate conidia, with a few distosepta, rounded or coronate at the apex, seceding schizolytically from monoblastic, integrated, terminal, annellidic or rarely sympodially extending conidiogenous cells. Ma et al. (2012) revised *Sporidesmiella* and accepted 26 species, based on the characters of proliferations of conidiogenous cells and conidial shape, size range and septation. Subsequently, 12 additional taxa have been added to *Sporidesmiella*, based on morphological characters, i.e. *S.curtiphora* (Monteiro et al. 2014), *S.bawanglingensis* and *S.nanlingensis* (Ma et al. 2015), *S.mammillata* (Heredia et al. 2015), *S.physconiicola* (Zhurbenko et al. 2015), *S.guangdongensis* and *S.jiangxiensis* (Ma 2016a), *S.lushanensis* and *S.jiulianshanensis* (Ma 2016b), *S.novae-zelandiae* (Hernández-Restrepo et al. 2018), *S.corniformis* (Ai et al. 2019) and *S.suttonii* (Kirk 2019). Recently, Luo et al. (2019) introduced a new species *S.aquatica* from freshwater habitats. Dong et al. (2021) reported a new species *S.obovioidia* from submerged wood. Up to now, 40 species have been accepted in *Sporidesmiella*.

So far, the molecular data of *Sporidesmiella* are relatively few; there are DNA sequences of only five species deposited in NCBI, i.e. *S.aquatic*, *S.fusiformis*, *S.hyalosperma*, *S.novae-zelandiae* and *S.obovoidia*. Therefore, most *Sporidesmiella* species have not been subjected to molecular phylogenetic analysis. Shenoy et al. (2006) classified *Sporidesmiellafusiformis* in the Melanommataceae according to the phylogenies with the combined LSU nu-rDNA and *RPB2* dataset. Luo et al. (2019), Crous et al. (2020) and Dong et al. (2021) accommodated *S.aquatic*, *S.hyalosperma*, *S.novae-zelandiae* and *S.obovoidia* within Junewangiaceae, based on the combination of LSU, ITS, *TEF1α* and *RPB2* sequences data. Therefore, as *Sporidesmiella* was suspected to be polyphyletic, the molecular data of the type species *S.claviformis* are in need of analysis.

Based on investigations of freshwater fungi in Jiangxi Province (Hu et al. 2012a, Huang et al. 2016, Hu et al. 2016, Song et al. 2018, Song et al. 2020), we reported a new species of *Sporidesmiella*, collected on submerged wood from freshwater habitats in Jiangxi Province. It was described and illustrated as *Sporidesmiellalignicola*, based on phylogenetic evidence of combined LSU, ITS, *TEF1α* and *RPB2* sequence data and morphological characters.

## Materials and methods

### Samples collection

Submerged wood samples were collected randomly from a stream in Xinfeng County, Ganzhou City, Jiangxi Province, China. The samples were taken to the laboratory in zip-lock bags and incubated in moist plastic boxes.

### Specimen examination

Fruiting bodies or colonies were examined following the method of Hu et al. (2012a) using a Nikon dissecting microscope. Samples were examined and photographed using a Nikon (Ni) compound microscope with differential interference contrast (DIC) (Hu et al. 2016). The fungal specimens were deposited in the Herbarium of Fungi, Jiangxi Agricultural University (HFJAU), Nanchang, China.

### Single spore isolation and cultivation

The fungal colonies on the rotten wood were picked up and placed in 200 μl sterile water to make a suspension, then the suspension was evenly spotted on potato dextrose agar (PDA), then cultured in a 28℃ incubator. The spore germination was observed every 12 hours and recorded. The germinating single spore was transfered to new PDA medium with a sterile needle under aseptic conditions and then cultured in a 28℃ incubator to obtain the pure strain.

### DNA extraction, PCR amplification and sequencing

DNA was extracted from the pure cultures with the CTAB method, following Doyle and Doyle (1987). Four gene regions, LSU, ITS, *TEF1α* and *RPB2* were amplified using the primer pairs LR0R/LR5, ITS1/ITS4, EF1-983F/EF1-2218R and RPB2-5F/RPB2-7cR, respectively (Vilgalys and Hester 1990, White et al. 1990, Liu et al. 1999). The amplification was performed following the method described by Hu et al. (2012b). The PCR products were examined using 1% agarose electrophoresis gels, stained with GelRed and purified and sequenced with the same primers at Tsingke Biotechnology Co. Ltd.

### Phylogenetic analyses

Four novel sequences (OK091615, MZ613187, OK323223, OK323222) from the new taxon, together with reference sequences obtained from GenBank (Table 1), were aligned with MAFFT version 7 (https://mafft.cbrc.jp/alignment/software/, Katoh and Standley 2013). The ML analyses were conducted with RAxML v. 7.2.6 (Stamatakis and Alachiotis 2010), using a GTRGAMMA substitution model with 1000 bootstrap replicates. The robustness of the analyses was evaluated by bootstrap support (MLBS).

The multilocus sequences were concatenated with PhyloSuite v. 1.2.2 (Zhang et al. 2020). The concatenated aligned datasets were analysed separately using Maximum Likelihood (ML) and Bayesian Inference (BI). ModelFinder (Kalyaanamoorthy et al. 2017) was used to select the best-fit model using AICc criterion. The best-fit model according to AICc was GTR+F+I+G4. Bayesian Inference phylogenies were inferred using MrBayes 3.2.6 (Ronquist et al. 2012) under partition model (2 parallel runs, 2,000,000 generations), in which the initial 25% of sampled data were discarded as burn-in. Modification of the final phylogenetic tree was done in FigTree v. 1.4.3 and Adobe Illustrator CS6.

## Taxon treatments

### Sporidesmiella lignicola

X.H. Li, H.Y. Song & D.M. Hu sp. nov.

82C931EC-2E83-5B9E-B19F-3B86D2F0180D

841439

#### Materials

- **Type status:** Holotype. **Occurrence:** recordedBy: Xiao-Hong Li; individualCount: 1; **Taxon:** taxonID: urn:lsid:biosci.ohio-state.edu:osuc_names:275502; scientificName: *Sporidesmiellalignicola*; acceptedNameUsage: *Sporidesmiellalignicola* X.H. Li, H.Y. Song & D.M. Hu, 2021, sp. nov.; parentNameUsage: Sporidesmiella P.M. Kirk 1982; kingdom: Fungi; phylum: Ascomycota; class: Dothideomycetes; order: Pleosporales; family: Junewangiaceae; genus: Sporidesmiella; specificEpithet: *lignicola*; taxonRank: species; verbatimTaxonRank: species; scientificNameAuthorship: X.H. Li, H.Y. Song & D.M. Hu; **Location:** continent: Asia; country: China; stateProvince: Jiangxi; county: Xinfeng; municipality: Ji'an; locality: Jinji Forest Farm; verbatimElevation: 305 m; locationRemarks: label transliteration: "Jiangxi, Jinji Forest Farm, 2020.7.7, Li Xiao-Hong"; [江西赣州市信丰县金鸡林场，2020年7月7日，李小红]; verbatimCoordinates: 25.4732 N, 115.2048 E; decimalLatitude: 25.4732; decimalLongitude: 115.2048; georeferenceProtocol: label; **Identification:** identifiedBy: Xiao-Hong Li and Dian-Ming Hu; dateIdentified: 2020; **Event:** samplingProtocol: collecting; eventDate: 07/07/2021; habitat: Freshwater; **Record Level:** type: PhysicalObject; language: en; rightsHolder: Dian-Ming Hu; institutionID: HFJAU 10001 (Dried culture with conidia); collectionID: FF019; institutionCode: the Herbarium of Fungi, Jiangxi Agricultural University (HFJAU); collectionCode: Fungi; ownerInstitutionCode: the Herbarium of Fungi, Jiangxi Agricultural University (HFJAU); basisOfRecord: PreservedSpecimen

#### Description

**Figure 1.**
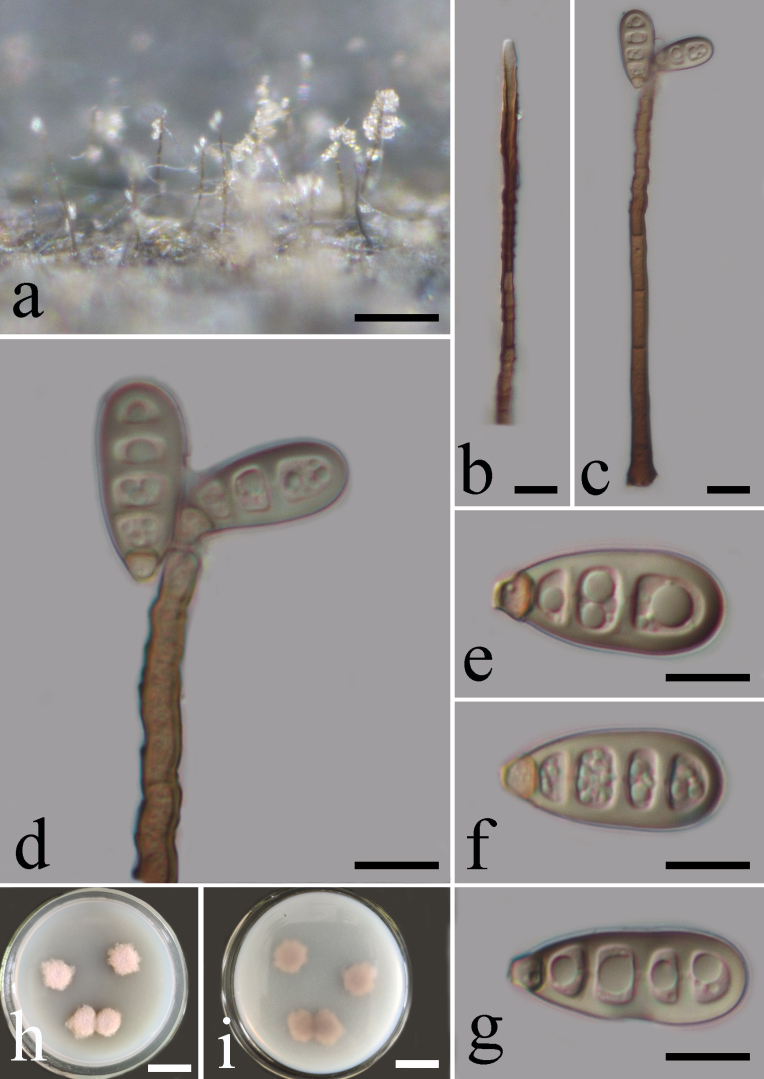
***Sporidesmiellalignicola*** (HFJAU 10001, Holotype) **a** Colony on wood; **b, c** Conidiophores; **d** Conidiophores with production of conidia; **e**–**g** Conidia; **h, i** Colony on PDA for 21 days (left-front, right-reverse). Scale bars: a = 125 µm, b–c = 12.5 µm, d–g = 10 µm, h–i = 1.5 cm.

Saprobic on decaying wood submerged in freshwater habitats. Colonies effuse, hairy, pale brown. Mycelium mostly superficial, partly immersed, consisting of unbranched, septate, smooth, thick-walled, brown to dark brown hyphae. **Sexual morph**: Undetermined. **Asexual morph**: Conidiophores 110–150 × 3–7 μm (mean = 124.6 × 4.2, n = 20), macronematous, mononematous, solitary, pale brown, smooth at the bottom and verrucose at the apex, septate, unbranched, straight or slightly flexuous. Conidiogenous cells 15–26 × 2–5 μm (mean = 22.4 × 4, n = 20), holoblastic, polyblastic, integrated, terminal, pale brown, cylindrical. Conidia 18–26 × 7–11 μm (mean = 21 × 8.9, n = 20), acrogenous, dry, cylindrical, narrowly clavate, obovoid to broadly obovoid to cuneiform, truncate at the base, rounded or rarely coronate at the apex, 2–3-distoseptate, pale olivaceous to olivaceous brown or brown, smooth. Conidial session schizolytic (Fig. 1).

**Figure 2.**
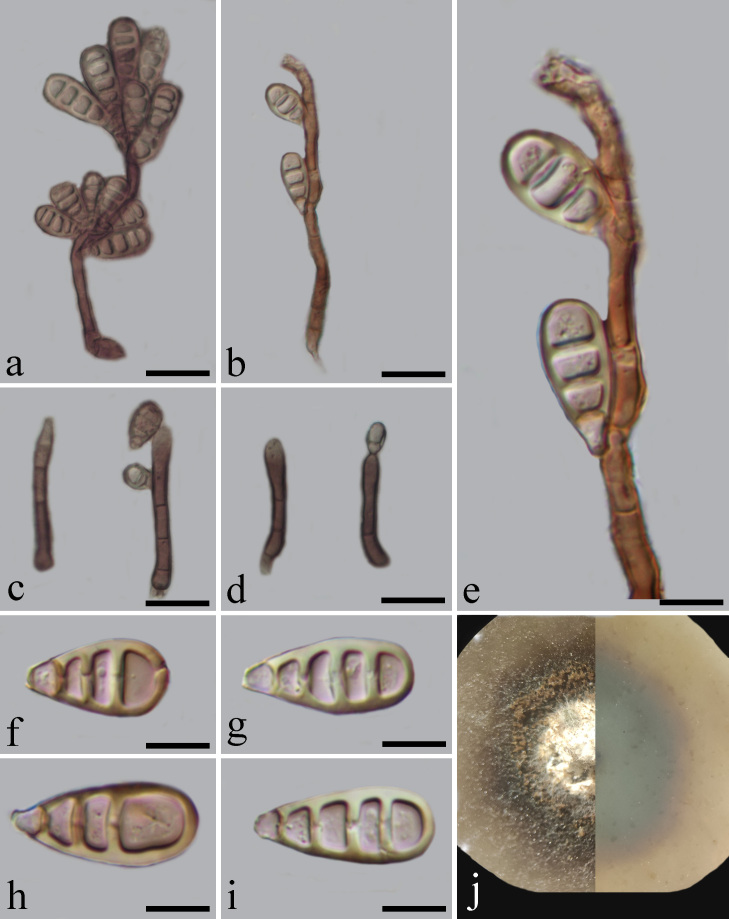
**a–b, e** Conidiophores with production of conidia; **c–d** Conidiophores; **f–i** Conidia; **j** Colony on PDA after 6 months (left-front, right-reverse). Scale bars: a–d = 25 µm, e–i = 10 µm, d–g = 10 µm.

**Culture characteristics**: On PDA, colony reaching 12 mm in 21 days at 28°C, pink from above, pink-grey from below, surface rough, dry, with loose mycelium and irregular edge. After half a year, the colony produces spores. The hyphae penetrate into the PDA medium, the surface colour becomes brown to dark brown, raised with white in the middle, reverse of culture pale brown to dark brown, with entire and regular edge. Mycelium composed of septate, pale brown, unbranched, smooth hyphae. Conidiophores macronematous, solitary, cylindrical, straight or slightly flexuous, septate, brown, smooth, thick-walled, 37–54 × 3.5–5.5 μm (mean = 46.5 × 4.6, n = 20). Conidiogenous cells holoblastic, polyblastic, integrated, terminal, pale brown, cylindrical, 10–26 × 3–7 μm (mean = 27.1 × 4.6, n = 20), slightly enlarged towards the apex. Conidia acrogenous, cylindrical, broadly obovoid to cuneiform, truncate at the base, rounded at the apex, 3–4-distoseptate, brown to pale olivaceous brown, smooth, 18–28 × 8–12 μm (mean = 22.3 × 9.6, n = 20) (Fig. 2).

#### Etymology

The specific epithet “*lignicola*” (Latin) meaning ‘‘growing on wood’’.

#### Ecology

Saprophyte on wood submerged in a small stream.

#### Notes

**Figure 3.**
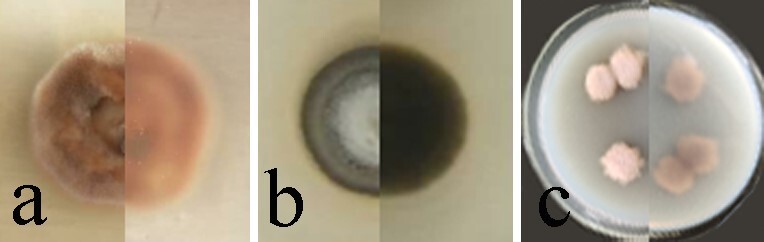
Comparisons of colonies on PDA (left-front, right-reverse) in *Sporidesmiellalignicola* and similar species. **a** *S.hyalosperma*; **b** *S.obovoidia*; **c** *S.lignicola.*

*Sporidesmiellalignicola* is characterised by being cylindrical, broadly obovoid to cuneiform, truncate at the base, rounded at the apex, 3–4-distoseptate, pale olivaceous brown to brown, smooth, which is consistent with the characteristics of *Sporidesmiella*. *Sporidesmiellalignicola* is similar to *S.obovoidia* and *S.hyalosperma* in having polyblastic conidiogenous cells and obovoid, 3–4-distoseptate, brown conidia (Luo et al. 2019, Dong et al. 2021). However, *S.lignicola* differs from other species in having longer and verrucose conidiophores (Table 2). In addition, the colonies of *S.lignicola* are pink from above, pink-grey from below, characteristics which were not observed in the other two species (Fig. 3, Table 2).

**Figure 4.**
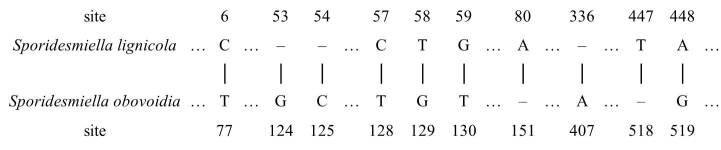
The specific base differences between *S.lignicola* and *S.obovoidia* in ITS. Different base pairs have been marked on specific sites, and the same base is omitted.

**Figure 5.**
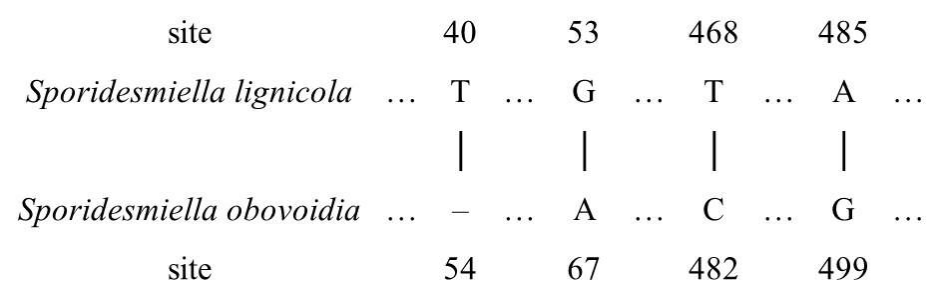
The specific base differences between *S.lignicola* and *S.obovoidia* in LSU. Different base pairs have been marked on specific sites, and the same base is omitted.

Based on a BLAST of NCBI’s GenBank nucleotide database, the most similar sequence was *Sporidesmiellaobovoidia*. The nucleotide comparison between *S.lignicola* and *S.obovoidia* showed differences of 10 and 4 nucleotides in ITS and LSU sequence data, respectively (Fig. 4, Fig. 5), which supported them to be different species (Jeewon and Hyde 2016).

Unfortunately, the strain could not be successfully activated due to improper operation during preservation. When the original culture was retained for 6 months, the sporulation of mycelium could be observed under the microscope (Fig. 2). We deposited the dried culture as specimens (HFJAU 10001) of this species.

## Analysis

### Phylogenetic analyses

**Figure 6.**
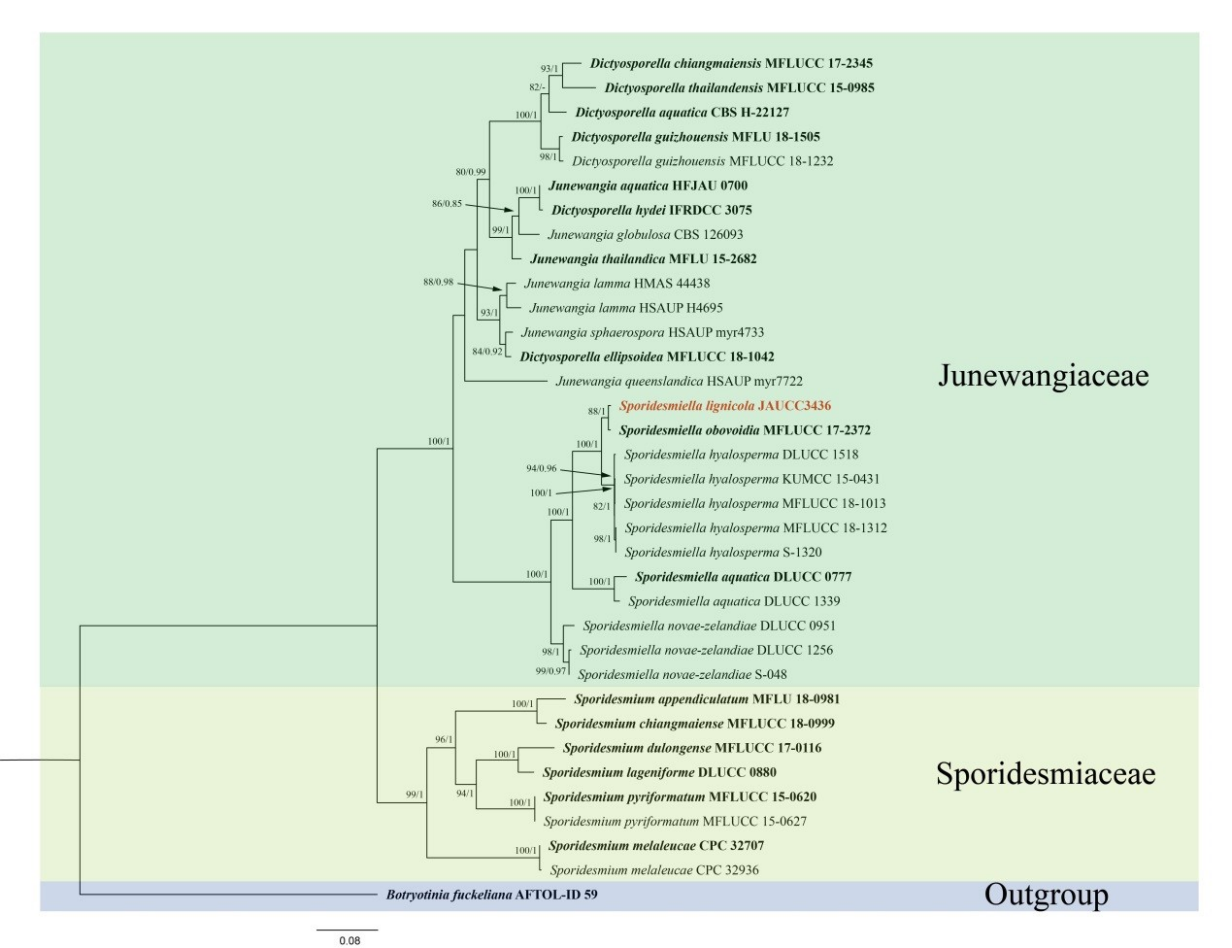
Phylogenetic tree inferred from a Maximum Likelihood analysis, based on a concatenated alignment of LSU, ITS, *TEF1α* and *RPB2* sequences of 35 strains representing *Sporidesmiella* species and other similar species. Bootstrap support values (ML) for Maximum Likelihood higher than 80% and Bayesian posterior probabilities (PP) greater than 0.80 are given at the nodes as ML/PP. The root of this tree is *Botryotiniafuckeliana*. Ex-type strains are in **bold**; new species are highlighted in red.

The analysed dataset comprised 35 taxa retrieved from GenBank and we selected *Botryotiniafuckeliana* (AFTOL-ID 59) as the outgroup taxon (Table 1). Partial nucleotide sequences of LSU (844bp), ITS (598bp), *TEF1α* (881bp), *RPB2* (1059bp) and, for a total of 3382 characters including gaps, were used to determine the phylogenetic placement of the new taxon. The generated ML and Bayesian trees were similar in topology and the best scoring RAxML tree is presented in Fig. 6.

The phylogenetic tree demonstrated that the new taxon (*Sporidesmiellalignicola*), together with species of *S.obovoidia*, *S.hyalosperma*, *S.aquatica* and *S.novae-zelandiae*, formed a distinct clade representing the genus *Sporidesmiella* with strong bootstrap support (100% MLBS, 1.00 PP). Additionally, in our phylogenetic analysis, the three genera *Dictyosporella*, *Junewangia* and *Sporidesmiella* constituted a well-supported clade with strong ML and BYPP bootstrap support (100% MLBS, 1.00 PP), which is in accordance with Luo et al. (2019) and Dong et al. (2021). *Sporidesmiellalignicola* appeared closely related to *S.obovoidia* and *S.hyalosperma*. Although *S.lignicola* (JAUCC 3436) clustered together in *S.obovoidia* (MFLUCC 17-2372) with high support (88% MLBS, 1.00 PP), they are not phylogenetically identical.

## Discussion

Kirk (1982) established the genus *Sporidesmiella* with *S.claviformis* as the type species, which had accommodated 40 species before this study. This study introduced *Sporidesmiellalignicola* as a new hyphomycetous fungus from freshwater habitats. In our phylogenetic analysis, *S.lignicola* clustered in *Sporidesmiella*, together with *Dictyosporella* and *Junewangia* forming a well-supported clade representing Junewangiaceae.

Many species of *Sporidesmiella* are found on decaying leaves, wood, bark, dead branches, cane and culms. At present, only three species have been found on submerged wood. Our research provides a new freshwater fungus found on submerged wood for *Sporidesmiella* and we provide four new sequences data, enriching the molecular database of *Sporidesmiella*.

As a decomposer, lignicolous freshwater fungi play an important role in freshwater ecosystem and material cycles in nature. They are also important biological resources, which have great application potential. Lignicolous freshwater fungi are a great treasure of resources to be developed. Many unknown species are waiting for us to understand and explore.

## Supplementary Material

XML Treatment for Sporidesmiella lignicola

## Figures and Tables

**Table 1.** Taxa used in this study and their GenBank accession numbers. Ex-type strains are in **bold**; newly-generated sequences are highlighted with **underline**. **Abbreviation: MFLU**: the Herbarium of Mae Fah Luang University, Chiang Rai, Thailand; **MFLUCC**: Mae Fah Luang University Culture Collection, Chiang Rai, Thailand; **HFJAU**: Herbarium of Fungi, Jiangxi Agricultural University, Nanchang, China; **CBS**: Centraalbureau voor Schimmelcultures, Utrecht, The Netherlands; **HSAUP**: Herbarium of Department of Plant Pathology, Shandong Agricultural University, Taian, China; **HMAS**: Mycological Herbarium, Institute of Microbiology, Chinese Academy of Sciences, Beijing, China; Dali University Culture Collection, Yunnan, China; **JAUCC**: Jiangxi Agricultural University Culture Collection, Nanchang, China. **KUMCC**: Kunming Institute of Botany, Chinese Academy of Sciences, Kunming, China; **CPC**: Culture collection of Pedro Crous, housed at CBS.

Taxon	Voucher/Culture	GenBank accession numbers			
		LSU	ITS	*TEF1α*	*RPB2*
***Botryotiniafuckeliana***	**AFTOL-ID 59^T^**	**AY544651**	**DQ491491**	**DQ471045**	**DQ247786**
***Dictyosporellaaquatica***	**CBS H-22127^T^**	**KT241022**	**_**	**_**	**_**
***Dictyosporellachiangmaiensis***	**MFLUCC 17-2345^T^**	**MW287765**	**MW286491**	**_**	**_**
***Dictyosporellaellipsoidea***	**MFLUCC 18-1042^T^**	**MW287758**	**_**	**_**	**_**
***Dictyosporellaguizhouensis***	**MFLU 18-1505^T^**	**MK593605**	**MK593606**	**_**	**_**
*Dictyosporellaguizhouensis*	MFLUCC 18-1232	MW287760	MW286487	MW396646	**_**
***Dictyosporellahydei***	**IFRDCC 3075^T^**	**MG813161**	**_**	**_**	**_**
***Dictyosporellathailandensis***	**MFLUCC 15-0985^T^**	**MF374364**	**MF374355**	**MF370958**	**MF370952**
***Junewangiaaquatic***	**HFJAU 0700^T^**	**MG213737**	**MG213738**	**_**	**_**
*Junewangiaglobulosa*	CBS 126093	MH875535	MH864078	**_**	**_**
*Junewangialamma*	HSAUPH 4695	KU751883	KU999971	**_**	**_**
*Junewangialamma*	HMAS 44438	KU751882	KU999961	**_**	**_**
*Junewangiaqueenslandica*	HSAUPmyr 7722	KX033575	KU999984	**_**	**_**
*Junewangiasphaerospora*	HSAUPmyr 4733	KX033572	KU999981	**_**	**_**
***Junewangiathailandica***	**MFLU 15-2682^T^**	**MW287762**	**_**	**_**	**_**
*Sporidesmiellaaquatica*	DLUCC 1339	MK849844	_	MN194035	MN124524
***Sporidesmiellaaquatic***	**DLUCC 0777^T^**	**MK849843**	**MK828692**	**MN194034**	**_**
*Sporidesmiellahyalosperma*	DLUCC 1518	MK849842	MK828691	MN194033	MN124523
*Sporidesmiellahyalosperma*	KUMCC 15-0431	MK849841	MK828690	MN194032	MN124522
*Sporidesmiellahyalosperma*	S-1320	MK849840	MK828689	_	MN124521
*Sporidesmiellahyalosperma*	MFLUCC 18-1312	MK849839	MK828688	MN194031	MN124520
*Sporidesmiellahyalosperma*	MFLUCC 18-1013	MW287773	MW286499	MW396654	MW504070
***Sporidesmiellalignicola***	**JAUCC 3436^T^**	**OK091615**	**MZ613187**	**OK323223**	**OK323222**
*Sporidesmiellanovae-zelandiae*	DLUCC 0951	MK849847	MK828695	MN194037	MN124526
*Sporidesmiellanovae-zelandiae*	S-048	MK849846	MK828694	_	_
*Sporidesmiellanovae-zelandiae*	DLUCC 1256	MK849845	MK828693	MN194036	MN124525
***Sporidesmiellaobovoidia***	**MFLUCC 17-2372^T^**	**MW287766**	**MW286492**	**_**	**_**
***Sporidesmiumappendiculatum***	**MFLU 18-0981^T^**	**MW287774**	**MW286500**	**_**	**_**
***Sporidesmiumchiangmaiense***	**MFLUCC 18-0999^T^**	**MW287771**	**MW286497**	**_**	**_**
***Sporidesmiumdulongense***	**MFLUCC 17-0116^T^**	**MH795817**	**MH795812**	**MH801191**	**MH801190**
***Sporidesmiumlageniforme***	**DLUCC 0880^T^**	**MK849782**	**MK828640**	**MN194044**	**MN124533**
*Sporidesmiummelaleucae*	CPC 32936	MH327854	MH327818	_	_
***Sporidesmiummelaleucae***	**CPC 32707^T^**	**MH327853**	**MH327817**	**_**	**_**
*Sporidesmiumpyriformatum*	MFLUCC 15-0627	KX710143	KX710148	MF135663	MF135650
***Sporidesmiumpyriformatum***	**MFLUCC 15-0620^T^**	**KX710141**	**KX710146**	**MF135662**	**MF135649**

**Table 2.** Comparisons of *Sporidesmiellalignicola* and similar species.

Species	Conidiophores (µm)	Conidiogenous cell (µm)	Conidia			Colour of cultures	References
			Shape	Colour	Size (µm)		
*S.hyalosperma*	Smooth, 90–110 × 3.5–4.7	35–48 × 4–4.5	obovoid or broadly clavate	olivaceous brown	18.5–25 × 8–10.5	reddish-brown from above and below	Dong et al. 2021
***S.lignicola***	**Verrucose at the apex, 110**–**150 × 3**–**7**	**15**–**26 × 2**–**5**	**broadly obovoid to cuneiform**	**brown or olivaceous brown**	**18**–**26 × 7**–**11**	**pink from above, pink-grey from below**	**This paper**
*S.obovoidia*	Smooth, 80–125 × 3.5–4.5	10–40 × 3–4	mostly obovoid or broadly clavate	brown or olivaceous brown	20–25 × 9–11.5	grey-dark brown from above, black from below	Dong et al. 2021
